# Towards Outdoor Electromagnetic Field Exposure Mapping Generation Using Conditional GANs

**DOI:** 10.3390/s22249643

**Published:** 2022-12-09

**Authors:** Mohammed Mallik, Angesom Ataklity Tesfay, Benjamin Allaert, Redha Kassi, Esteban Egea-Lopez, Jose-Maria Molina-Garcia-Pardo, Joe Wiart, Davy P. Gaillot, Laurent Clavier

**Affiliations:** 1 Univ. Lille, CNRS, UMR 8520–IEMN, F-59000 Lille, France; 2 IMT Nord Europe, 59650 Villeneuve-d’Ascq, France; 3 Information Technologies and Communications Department, Universidad Politécnica de Cartagena, 30202 Cartagena, Spain; 4 Chaire C2M, LTCI, Télécom Paris, Institut Polytechnique de Paris, 91120 Palaiseau, France

**Keywords:** EMF exposure, conditional generative adversarial network, optimization

## Abstract

With the ongoing fifth-generation cellular network (5G) deployment, electromagnetic field exposure has become a critical concern. However, measurements are scarce, and accurate electromagnetic field reconstruction in a geographic region remains challenging. This work proposes a conditional generative adversarial network to address this issue. The main objective is to reconstruct the electromagnetic field exposure map accurately according to the environment’s topology from a few sensors located in an outdoor urban environment. The model is trained to learn and estimate the propagation characteristics of the electromagnetic field according to the topology of a given environment. In addition, the conditional generative adversarial network-based electromagnetic field mapping is compared with simple kriging. Results show that the proposed method produces accurate estimates and is a promising solution for exposure map reconstruction.

## 1. Introduction

Wireless communication systems have become an inherent part of our daily life. Hence, monitoring the status of wireless systems phenomena, such as radio-frequency electromagnetic field (RF-EMF) exposure, is of great significance. In urban environments, there are many sources of EMF, including WiFi, 2G, 3G, 4G, and 5G mobile communication technologies. Although 5G promises many improvements compared to previous generations [1,2,3], there is growing anxiety regarding implementing the 5G network for two main reasons. The first one is that the frequency used is higher than the other mobile communication generations. The second reason is the ultra-dense base station implementation requirement. Several organizations, such as the International Commission on Non-Ionizing Radiation Protection (ICNIRP) and the Institute of Electrical and Electronics Engineers (IEEE), have conducted research on human exposure standards for EMFs, as mobile devices and base stations emitting EMFs for radio communication must comply with regulatory human exposure levels for EMFs [4,5].

Although sensor networks and on-site measurements are essential, they are confined systems that only allow a limited amount of EMF exposure monitoring. Locations of base stations and mobile devices in an urban setting are influenced by elements including building topology, roadways, vehicles, and urban city topology. To evaluate RF-EMF exposure, a power map must be constructed while taking these relevant factors into account. The challenge is reconstructing the EMF exposure map in an urban area from only a few sparsely located sensor-measured power values changing over time according to environment topology and network activity.

In this work, we aim to assess the RF-EMF exposure using only 50 fixed receivers sparsely located in a 1 km square urban environment, specifically in Lille city center, France. This is achieved using a conditional Generative Adversarial Network (cGAN) [6] architecture where the city topology is used as a conditional input. The proposed Exposure Map Generative Adversarial Network (EMGAN) method is an innovative approach inspired by the cGAN architecture. The EMGAN model learns and then estimates the features of outdoor wireless propagation, including diffraction, shadowing reflection, and the impact of building walls, materials, roadways, and city topography.

Additionally, other reconstruction techniques, such as kriging, are developed to evaluate and compare the with the proposed model. In this work, the unlicensed vehicular frequency band around 5.89 GHz is used as an arbitrary frequency to generate exposure maps. It should be highlighted that the proposed cGAN approach can be naturally extended to study the EMF exposure of any technologies (or combinations of several) across different frequency bands.

The main contributions of this paper can be summarized as follows:

- Generating a new simulated dataset called “LilleExposureMap”, which consists of EMF exposure maps in Lille, France.
- Develop the generator and discriminator for the proposed EMGAN utilizing the deep convolutional structure and auto-encoders analogy to learn about signal propagation and calculate the map of EMF exposure.

The goal of our work is to reconstruct maps based on a limited number of sensors’ measures without the knowledge of the base station activity. To train the generative model, real samples are needed but we cannot have fully measured maps. To replace them, we use a ray-tracing-based simulator of a cellular network (VENERIS). When running, our solution does not rely on the knowledge of the network activity or position of transmitters and receivers, but only a few measured points. This significantly differs from a network propagation simulator such as VENERIS, which calculates the field based on the transmitter location, antenna pattern, and network activity.

The performance of the designed EMGAN is evaluated and shows a good reconstruction despite a reduced number of fixed sensors. The paper is organized as follows. In Section 2, the review of related works is discussed. In Section 3, the data set is described. Section 4 describes the proposed EMGAN model. The results and findings are presented and discussed in Section 5. Section 6 concludes the work.

## 2. Related Work

Accurate radio frequency power map estimation is computationally expensive in a geographic region. Predicting power coverage in urban areas typically requires ray-tracing and also empirical/semi-empirical models, such as close-in (CI), floating intercept (FI), alpha, beta, gamma (ABG) [7,8,9], etc., simulations to determine how radio signals propagate and are distributed over an area. Deterministic and empirical models are used for propagation prediction in earlier studies. Some examples are the dominant path model [10], ray-tracing [11,12], and empirical models, such as [13]. Ray-tracing (RT) techniques offer the highest degree of precision, but often at the expense of high processing demands and a reliance on the accuracy of the tridimensional (3D) model of the investigated scenario [14]. RT techniques describe the propagating field as a series of propagating rays that reflect, diffract, and scatter over various environmental components. This high-frequency approximation (optical ray) to the Maxwell equations is the foundation for these approaches. This methodology has often proven computationally too expensive to be employed in large and, in particular, dynamic contexts. Hence, network simulators only provide stochastic or streamlined hybrid approaches [15]. If they are well suited to the coverage maps, they can not be used for exposure maps, which would require the simulation of many different technologies and the consideration of the number and positions of active users.

Data-driven interpolation methods presume that some measurements are given at specific locations. These methods predict the function at non-measured locations via some signal processing approach, e.g., kriging [16] relies on a model of the physical characteristics. The work in [17] used kriging to perform spatial interpolation of climate data. The method works well when significant measurement points are considered. In [18], kriging is applied to spatially reconstruct the EMF in an indoor scenario. However, the authors concluded that the reconstruction performance of the model could be improved by increasing the measurement points.

In [19], the authors use a convolutional neural network (CNN)-based Generative Adversarial Network (GAN) [20] to estimate the power spectrum map while considering urban cognitive radio networks. The employed bandwidths are 25 MHz and 75 MHz, and a uniform distribution of users was assumed. The under-sampled power spectrum maps were used as input for the image reconstruction task using a GAN model based on an autoencoder analogy. However, the authors used the inverse polynomial law model in calculating power spectrum maps (PSMs) to depict propagation characteristics. Additionally, the authors did not consider the topology as conditional input.

For estimating radio maps, two recent papers proposed deep learning approaches [21,22], where the authors implemented a CNN [23] as the mapping function to estimate the radio map for each Tx-Rx location. Every trained network defines a specific map, and a new city map is needed to train the network. Other works on power map prediction use fully connected neural models, which do not consider the city topology as additional information [24,25,26]. In addition, the authors in [27] used a convolutional autoencoder [28] network for spectrum map interpolation, where several transmitters with unknown locations operate. Buildings were superimposed on the estimated map after the model inference. So far in the literature, all the works did not consider the propagation characteristics of the area of interest, which can help to make the accurate mapping. However, in the proposed method, the city topology is considered a conditional input to the cGAN model to represent the urban environment characteristics. To the best of our knowledge, no study in the literature considers the environment topology as additional input.

## 3. Dataset and Simulator

**Figure 1:**
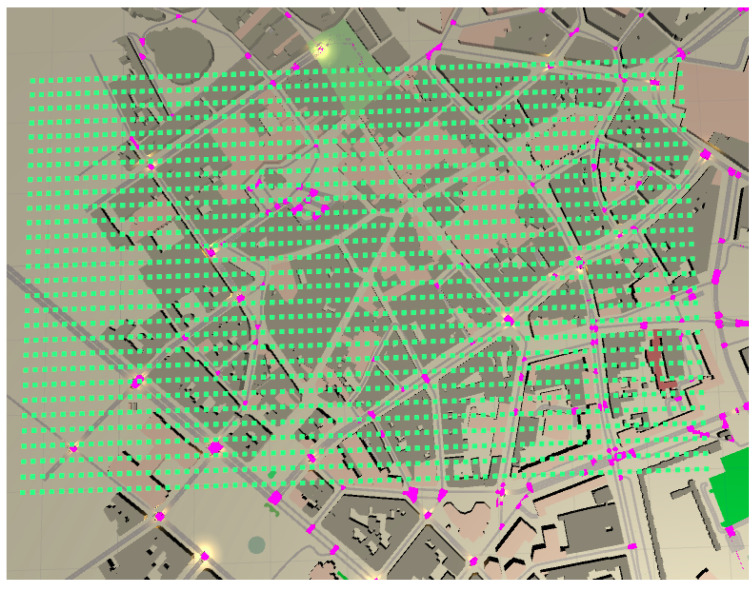
Three-dimensional environment model of Lille City $1\;{\mathrm{km}}^{2}$ area with 2088 receiver grid represented in green squares.

This work presents a new dataset, called “LilleExposureMap” of 6006 simulated EMF exposure maps in Lille, France, obtained from Veneris-Opal. Different moving transmitter positions are used in order to generate different maps. The considered number of simulated EMF exposure maps is fixed to minimize the computational complexity. One km square area is selected where three different simulations are performed, placing three different moving positions of transmitters in the area of interest (see Figure 1). The method is suitable for any technology (1G, 2G, 3G, 4G, 5G, and so on), heterogeneous networks, and frequencies. In Veneris-Opal simulations, when transmitters or receivers move, Veneris-Opal updates the positions before launching new rays and computing the received power so it can cope with scenarios with mobility. Here, we consider a static environment such that no mobility between the transmitter and receivers are taken into account at a certain time step. To generate the data of different full maps, the transmitter is set to move in one direction so that the data changes at different time steps.

The 3D environment model is implemented in ‘VENERIS-OPAL’ an open-source ray tracing network simulator [29]. Veneris-Opal is a simulation framework for research on vehicular networks and Cooperative Automated Driving. However, it can also be used for general wireless network simulation, which needs 3D environment-aware propagation simulation. On top of the Unity game engine [30], it includes a realistic vehicular model and a set of driving and lane change behaviors that reproduce the traffic dynamics integrated using SUMO [31], a ray-launching GPU-based propagation simulator called Opal [32] and a set of modules that enable bidirectional coupling with the widely used OMNET++ network simulator.

A map of Lille city center from OpenStreetMap [33] is used for the ray launching simulations in Veneris-Opal. More specifically, the dataset contains objects with rough surfaces like 3D building walls, statues, roads, etc., interacting with the rays in complex ways. Moreover, gaps in the object, e.g., bridges and rays at different levels, can pass or reflect from such objects. The presence of the object in the environment, and several ray interactions result in a complex power distribution representing a real-life environment. The transmitters are assumed to use an isotropic antenna, producing omnidirectional radio waves. The height and power of the transmitting antenna were set at a maximum of 15 m and 20 W, respectively. OpenStreetMap does not provide the height of most buildings. Only some buildings have this information. The height of the remaining buildings was set to 10 m in the simulator. The propagation model in ray launching considers reflections on 3D buildings, resulting in more complex patterns. Even if VENERIS can handle a very high number of iterations, a convergence study shows that two interactions are enough for reliable results in this kind of environment.

For simulations, three transmitters are used at 5.89 GHz with 20 W transmitting power, and other simulation parameters are used, such as azimuth, elevation, reflection, etc. To generate the real exposure map images, 2088 receivers at the height of 1.5 m are placed in a uniform grid in the area of interest, as shown in Figure 1. The receivers inside the buildings are not considered. The goal of the paper is to reconstruct the outdoor exposure map. Thus, the receivers inside buildings have 0 values in simulations to generate data. There are approximately 1269 receivers in outdoor locations. Nonetheless, this number of points is sufficient to capture the dynamics of the field and train the model.

**Figure 2:**
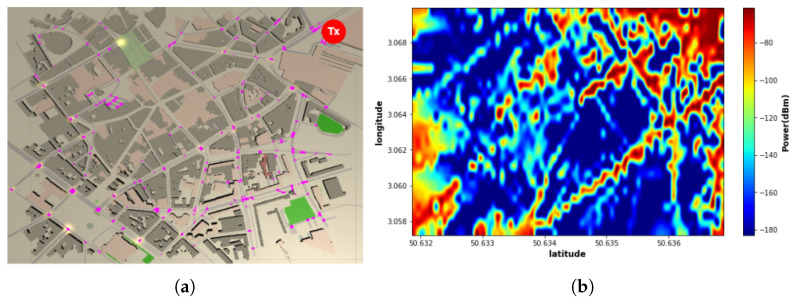
RF-EMF exposure reference map: (**a**) Transmitter’s location corner; (**b**) Transmitter at upper right corner.

Using Veneris-Opal, the received power in a dense urban environment by ray tracing is simulated and converted into images using Python. Then, it is employed as a reference map. Figure 2 gives an example of reference exposure maps when the transmitter is located at the top right corner. Each map has a dimension of 512 height, 512 width, and 3 RGB color channels.

**Figure 3:**
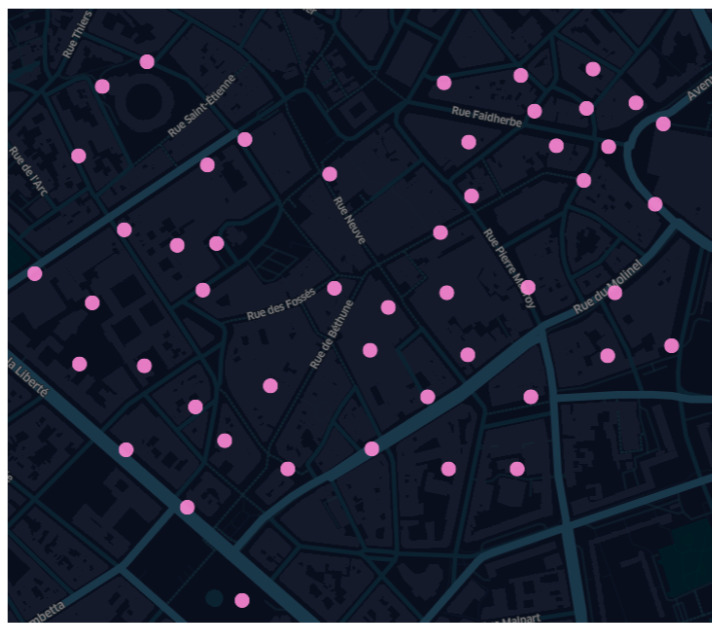
Fifty sparsely located sensor maps in the Lille city center. The dots represent the sensors.

The area of interest, Lille city center, is 1 km square, and 50 sensor points were selected from the reference map to generate the undersampled sensor map images. The locations of the sensors are constrained by the position of the lighting poles and are shown in Figure 3, and we consider 15, 30, and 50 fixed locations taken from the reference map images for generating the test set.

**Figure 4:**
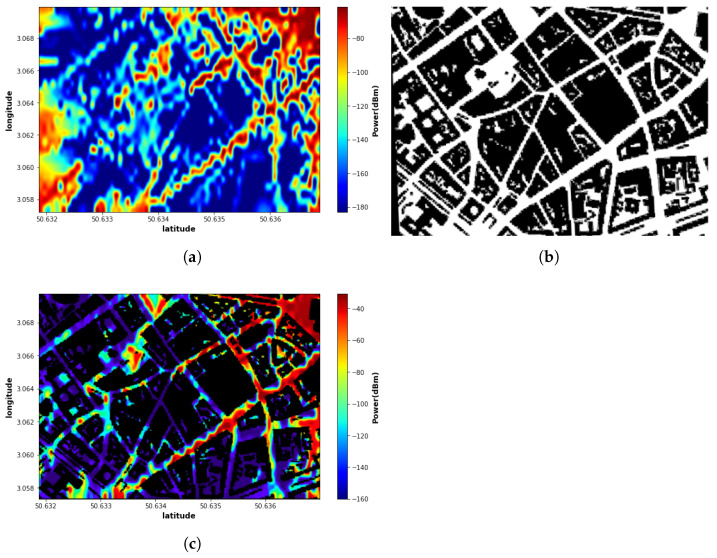
RF-EMF exposure reference map: (**a**) Reference map; (**b**) City topology; (**c**) Map superimposed on city topology.

To capture the city topology effect, we used an image where black and white represent buildings and roads, respectively (with no intermediate grey levels) as a conditional input to the cGAN network. The topology image is a black-and-white image of the environment. The generator of the cGAN model takes two images as input, one is the sparse sensor map, and the other is the topology image as conditional input where in the image, the black is the buildings where it has zero value for the buildings and white is the roads so the network can learn the buildings in the map for inference. An example of the topology image is shown in Figure 4b, and the EMF map with city topology overlaid is illustrated in Figure 4c.

It is important to note that less than 1% of the reference image area in terms of pixels is covered by sensors in the most optimistic case, i.e., when 50 measurements are taken into account.

## 4. The Proposed EMGAN Model

We propose a deep learning method inspired by a conditional GAN architecture adapted to image-to-image translation. Several studies have been conducted using this specific model architecture [34,35,36] for different applications. For instance, in [37], U-net based model known as the EME-Net model is proposed to map the EMF. EME-NET is a deterministic model, whereas EMGAN is a probabilistic model. In both approaches, the U-net model is used but for different purposes. The U-net model is used as a predictive model in EME-Net, and as the generator in the proposed EMGAN approach. In addition to the generator, the EMGAN model uses a discriminator. The nature of using a discriminator model is certainly the cause. It offers a loss value that reflects both the output-target discrepancy at the pixel level and the more profound comprehension of the images that result from this. The discriminator will therefore provide a signal that favors the generation of more genuine images by learning about the real distribution of the data.

In this work, the network learns to estimate the propagation of an electromagnetic field according to a distribution of sensors. Our model is conditioned by a map that represents the topology of a city, thus forcing the model to adapt to a targeted topology, whether indoor or outdoor. We also expect that the model can extend in indoor configurations, probably requiring an adapted learning phase. In the following section, the input and output data are presented in Section 4.1 and then the proposed network architecture is discussed in Section 4.2.

### 4.1. Input and Output Data

To train the model, input data for the generator are the sensor map and one city topology image with a dimension of 512×512×3. The output is a fake full-exposure map image. For the discriminator, inputs are the generated fake images from the generator and the real full exposure map image simulated by Veneris-Opal with a dimension of 512×512×3. The output is classified as whether the data is fake or real, estimated full exposure map. The discriminator uses the information from the sensors through the fake map from the generator and compares this fake map with the Veneris exposure map considered as the real map.

### 4.2. Network Architecture

**Figure 5:**
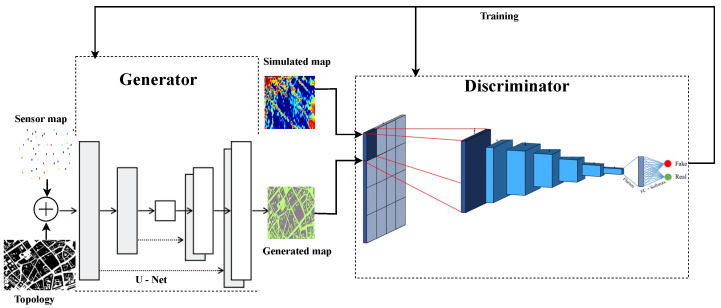
Proposed conditional GAN.

In this section, the proposed network architecture is presented. As shown in Figure 5, the proposed model consists of a U-Net-based Generator and a CNN-based Discriminator, where detailed descriptions of the network are given in Section 4.2.1 and Section 4.2.2.

#### 4.2.1. U-Net Generator

Estimating the EMF exposure map is an image-to-image translation task. The model’s inputs consist of two images, a sparse sensor measurement map, and the city topology as a conditional input to the generator, represented as a three-dimensional matrix (height, width, and color channel). The U-net [38] based generator is used in the proposed model. Olaf Ronneberger et al. created the U-NET for biomedical image segmentation. The design has two contraction routes, which are also known as encoders, that are used to capture the information of an image. We use downsampling in the encoder, which is basically a conventional stack of convolution and max pooling layers. The second path, known as the decoder, is the expansion path, which is symmetrical to our contraction and serves to make it possible to precisely localize objects using transposed convolutions. The details of the model are given in the following subsections.

Three channels, red, green, and blue, are combined to create a picture [39]. A channel represents the color and color intensity of an image. The proposed method reconstructs the final image using a three-dimensional image tensor with three channels. The input sensor measurement map is sparse because each pixel’s color intensity corresponds to a sensor-measured value at that location.

Since estimating the EMF exposure map is mainly an image-to-image translation task, the model’s inputs are two images, a sparse sensor measurement map, and the city topology as a conditional input to the generator (the U-net model) represented as a three-dimensional matrix, (height, width, and color channel). An image is built by combining three channels, i.e., red, green, and blue [39]. Simply put, a channel refers to color intensity and color in the image. In contrast, a three-dimensional image tensor with channel depth three is utilized for our method to reconstruct the output image. The color intensity of pixels of the input image represents the sensor-measured value at a corresponding location, making the input sparse sensor measurement map.

The generator is an encoder-decoder model using a U-Net architecture. The model takes a source image (e.g., a sensor map) along with a city topology image as a conditional input and generates a target image (e.g., a complete exposure map with the effect of topology). It does this by first downsampling or encoding the input image down to a bottleneck layer, then upsampling or decoding the bottleneck representation to the size of the output image. Moreover, skip connections are added between the encoding and corresponding decoding layers for learning features from input images. The model is built to learn more intricate wireless propagation aspects of the target area and translate it to an EMF map for exposure assessment.

**Encoder**: The sensor map is the input to the encoder’s input layer. The decoder module consists of several blocks, each of which has the following setup:

- Using a kernel size of 3×3 and a stride of 1, two convolutional layers are applied in succession. The input layer uses tensors of a size of 512×512×3, which represent a three-dimensional sensor map picture. This results in new dimensions with 16 channels and raises the feature map’s channel count.
- The rectified linear unit (ReLU) is the activation function that is being used. This function enables us to take only positive values after convolution operation.
- A max-pooling layer connects previous layers. This layer downsamples the feature map by taking the biggest value in each patch of each feature map. This creates new dimensions of 64×64×16.

The first block’s layers are repeated in the decoder module, where the depth or channel number rises to 8×8×512 and the size of the feature map rapidly decreases.

**Decoder**: Five symmetric reduction module blocks are employed in the decoder module along with a transposed convolutional layer for upsampling. The feature map’s height and width are set in the layer’s properties to be doubled, but the depth (number of channels) is set to be decreased by half. For the purpose of extracting more precise features from the feature map, two consecutive convolutions are used. The symmetric U-shaped generator model architecture contains five blocks on each module.

#### 4.2.2. Discriminator

The discriminator is a deep convolutional neural network in cGAN explicitly used for conditional image classification. It takes all three images as input, the sparse sensor map, the target image, and the conditional topology image. It estimates the likelihood of whether the target image is real or a fake translation of the source image having the effect from the topology as well. The effective receptive field of the model is the core of designing the discriminator. This is called a PatchGAN [36] model, which defines the connection between one output of the model to the number of pixels in the input image. The output estimate of each model corresponds to a 70×70 square or patch of the input image. This method has the advantage that the same model may be used to process input images that are bigger or less than 256×256 pixels. The size of the input image determines whether the discriminator’s output is a single value or a square activation map of values. Each value represents the chance that a patch in the input image is real. These values can be averaged if necessary to provide a global probability or categorization score.

### 4.3. Loss Functions

The loss function of the proposed cGAN model contains the discriminator and the generator part as shown in (1):

(1) $$L_{cGAN}\left(G,D\right)=E_{x,y}\left[\log D(x,y)\right]+E_{x,z}\left[1-\log D(x,G(x,z)\right]$$

where *x* is the input image, *y* is the output image, and *z* is the conditional image.

The generator *G* is not only trying to reduce the loss from the discriminator but also trying to move the fake distribution close to the real distribution by using the *L*1 loss given by (2):

(2) $$L_{L1}\left(G\right)=E_{x,y,z}\left[∥y-G(x,z)∥\right].$$

The loss function of the generator network is stated in (3):

(3) $$G^{★}=\arg\min_{G}\max_{D}L_{cGAN}\left(G,D\right)\lambda_{L1}\left(G\right).$$

## 5. Results

### 5.1. Training Set-Up

For the input training samples, 50 sensor measurement locations are used. Training parameters are listed in Table 1.

### 5.2. Evaluation Metrics

The structural similarity index (SSIM) [40] and peak signal-to-noise ratio (PSNR) are between the reconstructed map and the reference map in order to assess the model performance. Values between −1 and 1, where 1 denotes perfect resemblance, are provided by the SSIM model, which captures the observed change in the structural information of the picture. The PSNR test compares the distortion power to the greatest possible pixel intensity. The SSIM index is calculated on various windows of an image. The measure between two windows *x* and *y* of common size N×N is given below along with PSNR:

(4) $$\mathrm{PSNR}=10\log_{10}\times \frac{{\mathrm{MAX}}_{I}^{2}}{\mathrm{MSE}},$$

where ${\mathrm{MAX}}_{I}$ is the maximum possible pixel value of the image. When the pixels are represented using 8 bits per sample, this is 255. The degree of inaccuracy in statistical models is measured by the mean squared error or MSE. Between the observed and estimated values, it evaluates the average squared difference. The MSE is equal to 0 when a model is error-free. Its value increases when the model error does as well. The mean squared deviation is another name for the mean squared error (MSD).

(5) $$\mathrm{SSIM}(x,y)=\frac{\left(2\mu_{x}\mu_{y}+c_{1}\right)\left(2\sigma_{xy}+c_{2}\right)}{\left(\mu_{x}^{2}+\mu_{y}^{2}+c_{1}\right)\left(\sigma_{x}^{2}+\sigma_{y}^{2}+c_{2}\right)},$$

where, $\mu_{x}$ and $\mu_{y}$ are the pixel sample mean of *x* and *y*, $\mu_{y}$. $\sigma_{x}^{2}$ and $\sigma_{y}^{2}$ are the variance of *x* and *y*, $\sigma_{xy}$ is the covariance of *x* and *y*. $c_{1}={\left(k_{1}L\right)}^{2}$ and $c_{2}={\left(k_{2}L\right)}^{2}$ are the variables to stabilize the division with weak denominator, where *L* is the dynamic range of the pixel values. $k_{1}$ and $k_{2}$ are set to 0.01 and 0.03.

Finally, we will consider the pixel-to-pixel error that we will denote by *R*. To calculate *R*, in dB, given by:

(6) $$R\left(x,y\right)=10\log_{10}\left(\frac{x}{y}\right).$$

We will represent either the probability distribution of *R* or the cumulative distribution of $\left|R\right|$. This allows us to have a more detailed understanding of the error behavior.

### 5.3. Visual Analysis

**Figure 6:**
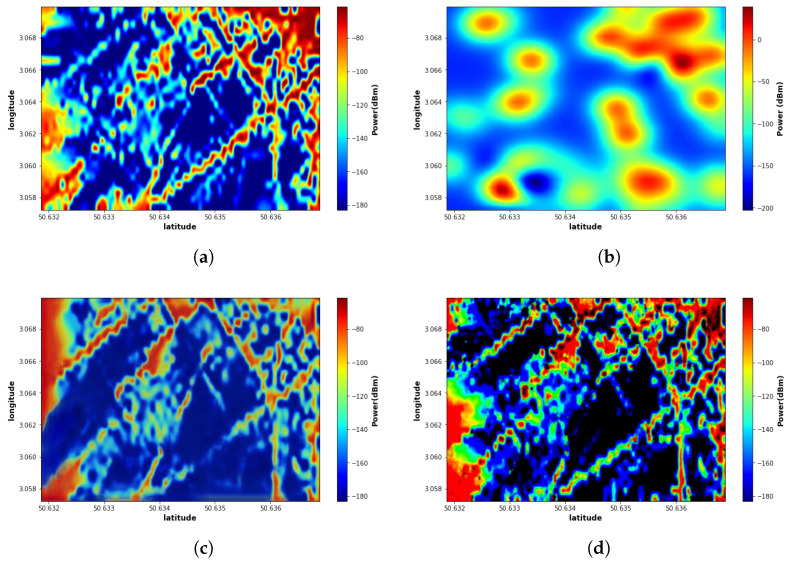
Comparison of Reconstructed maps of the proposed model and other different models: (**a**) Real map; (**b**) Simple kriging; (**c**) EME-Net model; (**d**) Proposed EMGAN model.

The proposed EMGAN model is compared with the EME-Net model in [37] and the kriging method when only 50 measurement points are considered. All models are trained and tested on the same training and test data sets. As illustrated in Figure 6, the proposed EMGAN model outperforms the kriging model. The EMGAN-based (Figure 6d) reconstructed map looks very close to the real map, and few details are missed by the EME-Net-based (Figure 6c), whereas the kriging-based (Figure 6b) encounters significant loss.

**Figure 7:**
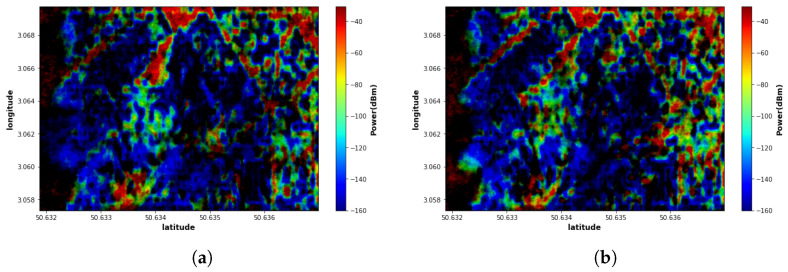
EMGAN-based reconstructed maps when different numbers of sensors are considered: (**a**) Using 15 sensor maps; (**b**) Using 30 sensor maps.

Additionally, the proposed EMGAN model performance is analyzed by varying the number of measurement points. Figure 7 shows the EMGAN-based reconstructed maps using 15 and 30 sensor measurement points. The figure illustrates that the performance of the proposed EMGAN model remains consistent even when a few measurement points are considered, although some degradation can be observed.

**Figure 8:**
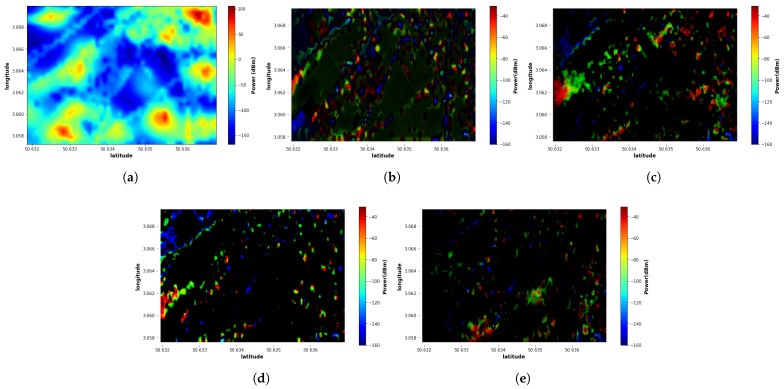
Error maps of the proposed EMGAN for different numbers of sensors and EME-Net model: (**a**) Kriging 50 sensors; (**b**) EME-Net 50 sensors; (**c**) EMGAN 15 sensors; (**d**) EMGAN 30 sensors; (**e**) EMGAN 50 sensors.

The error map between the reconstructed map and the real map is illustrated in Figure 8. The error map shows that the proposed EMGAN model (Figure 8c–e) has a significantly low error compared to the other models. The kriging approach exhibits very poor results. The approach is not well adapted to such a spatial undersampling. Further studies, though, should be performed to take into consideration the environment topology and the propagation models. However, this is a different approach and is out of the scope of this paper.

### 5.4. Quantified Analysis

**Figure 9:**
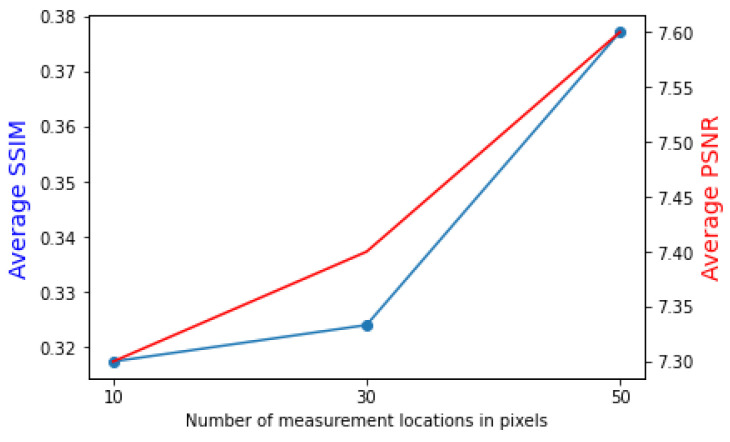
Average SSIM (in blue line) and PSNR (in red line) of the proposed EMGAN with a varying number of measurement points.

Figure 9 presents the averages of SSIM and PSNR as a function of the number of measurement points. As the number of measurement points rises, so do the averages of SSIM and PSNR. Since these metrics present the same trend, this indicates that the reconstruction process is coherent regarding similarity and image quality.

**Figure 10:**
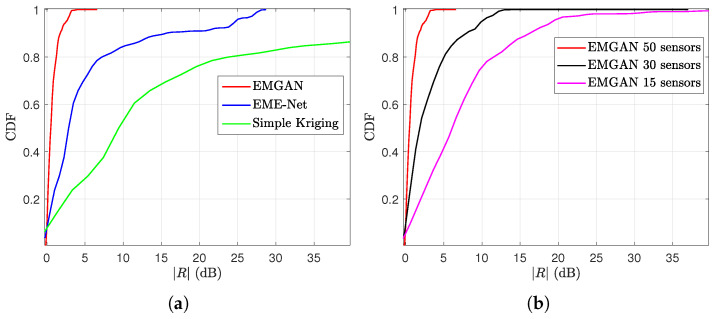
CDF of the models as a function of the absolute ratio |R| between the reconstructed map and real map: (**a**) Different models; (**b**) EMGAN with varying numbers of sensors.

The cumulative distribution function (CDF) of the error ratio *R* of the proposed EMGAN and other models is shown in Figure 10. As illustrated in Figure 10a, the proposed EMGAN model outperforms the kriging and EME-Net methods. In addition, Figure 10b shows that, despite the optimistic visual evaluation, the performance of the proposed EMGAN degrades as the number of sensors decreases. On the 1 km${}^{2}$ area we are studying, 50 sensors are needed to avoid some large deviations.

**Figure 11:**
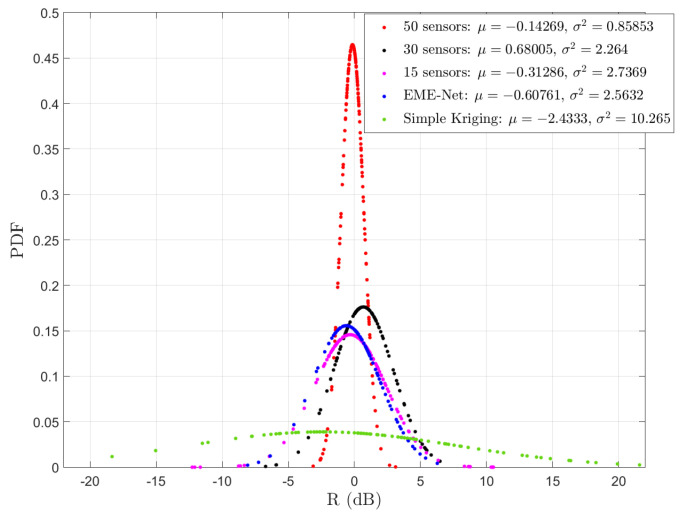
The probability density of the ratio R between the reconstructed map and real map when different numbers of sensors are used.

In Figure 11, the probability density function (PDF) of the ratio between the reconstructed maps and the real exposure map of the proposed EMGAN model with different numbers of sensors, EME-Net, and simple kriging methods are presented. The error ratio (in dB) distribution can be well approximated by a Gaussian random variable. We first notice that the mean is rather close to 0, meaning there is no significant bias in the prediction steps. The second important point is that we note the reduction in the variance with the increase in the number of sensors. With 50 sensors in the studied 1 km${}^{2}$ area, the variance is reduced to 0.85, which seems a reasonable value for an exposimetry study, resulting in more than 90% of the error ratio below 3dB. We also notice that the EME-Net approach with 50 sensors is not as good as the proposed approach with 30.

## 6. Conclusions

In this work, we present the EMGAN methodology, a deep learning-based exposure maps estimation method for urban environments. The generator and the discriminator are developed to estimate the exposure maps and improve the estimation accuracy by incorporating the city topology as a conditional input to the model. Instead of making direct, inaccurate, or biased assumptions about radio propagation, the EMGAN algorithm learns and uses radio environment information from the training process. In addition, it offers a highly accurate estimation performance, significantly more accurate than traditional approaches, according to experimental data. Future work will concentrate on expanding the estimation of exposure maps using EMGAN to the temporal dimension.

## Figures and Tables

**Table 1:** Training parameters.

Parameters	Value
Total number of images	6006
Input samples	2500
Test set	503
Optimizer	ADAM
Learning rate	$4\times 10^{-4}$
Batch size	2
Decay rate	$1\times 10^{-6}$
Epochs	4000

## Data Availability

Not applicable.
